# Point prevalence and sex-specific associated factors of depression in Latvian general population

**DOI:** 10.3389/fpsyt.2023.1065404

**Published:** 2023-03-28

**Authors:** Vineta Viktorija Vinogradova, Anda Kivite-Urtane, Jelena Vrublevska, Elmars Rancans

**Affiliations:** ^1^Department of Psychiatry and Narcology, Riga Stradins University, Riga, Latvia; ^2^Institute of Public Health, Riga Stradins University, Riga, Latvia; ^3^Department of Public Health and Epidemiology, Riga Stradins University, Riga, Latvia

**Keywords:** depression, epidemiology, prevalence, general population, sex-specific factors

## Abstract

**Background:**

This cross-sectional study aimed to determine the current prevalence of depression, and analyze sex-specific associated socio-demographic and health-related factors for depression in a representative sample of the general adult population of Latvia.

**Methods:**

Specially trained professional interviewers conducted computer-assisted face-to-face interviews with a multistage stratified probability sample from the general Latvian adult population (*n* = 2,687). A 9-item Patient Health Questionnaire (PHQ) was used for assessment of depression. Respondents were interviewed using the specially developed questionnaire about sociodemographic factors as well as the alcohol use disorder module of the Mini International Neuropsychiatric Interview. Binary logistic regression was used to calculate the odds ratios (OR) for the univariate and multivariate logistic analyses.

**Results:**

The point prevalence of depression according to the PHQ-9 was 6.4% (95% CI 5.8–7.6). After adjustment for all independent variables analyzed, being divorced, widowed, or living separately increased the odds of depression [aOR 2.6 (95% CI, 1.2–5.8), *p* = 0.02] in males. For females, unfinished primary education [aOR 5.2 (95% CI 2.0–13.6), *p* = 0.001] and economically inactive status [aOR 2.0 (95% CI, 1.1–3.6), *p* = 0.03] were strongly associated with depression.

**Limitations:**

The cross-sectional design of the study did not allow us to draw conclusions about causality. Patients with bipolar, organic, and symptomatic depression states were not excluded.

**Conclusion:**

The prevalence of depression in the general adult population is 6.4%, with the most significant sex-specific factors associated with depression for males – being divorced, widowed, or living separately, and for females it was poor education and economic inactivity.

## 1. Introduction

Mood disorders are the second most prevalent class of mental disorders, and major depressive disorder, in particular, is the most frequent mood disorder (1). The proportion of the global population with depression has been estimated to be 3.8% (2). Depression is a mental disorder that is frequently underdiagnosed and undertreated (3–6). Underdiagnosis of depression is also a concern in Latvia. The first data on the point prevalence of depression in Latvia were published in 2014 (7) by the participants of our research group. Data from a general population survey conducted in 2011 were used in this study. For approximately 10 years, the general population of Latvia has not been screened for depression, and there is a lack of up-to-date information on depression prevalence among adults. According to our previous research, the point prevalence of depression in the general population of Latvia is 6.7% (7), and the 12-month prevalence of major depressive disorder is estimated to be 7.9% (8), which means that at least 115,000 cases of depression should be registered. At the same time, data from the Latvian Center for Disease Prevention and Control show that in 2021, only 10,737 patients were diagnosed with a depressive episode or recurrent depressive disorder and registered in the National Health Service Register (9). This means that most cases of depression remain undetected, and patients do not receive adequate treatment. Depressive disorder, if left untreated, has a considerable impact on the public health system and is associated with an economic burden, composed of direct medical, indirect workplace, and suicide-related mortality costs (10). Patients with somatic diseases and untreated depression more often use secondary care facilities, leading to excessive and unwarranted expenses (6). Moreover, people with major depression are at risk of premature death (11). The life expectancy of patients with depression is approximately 7–10 years shorter than that of people in the general population, which can be explained by both somatic comorbidities (12, 13) and increased suicide risk (13, 14) in patients.

Although effective treatment for this disease is available, only a minority of people are being treated in conformity with the existing guidelines and receiving adequate treatment (15). As mentioned above, timely detection and treatment of depression is important for decreasing disability, prolonging life expectancy, and increasing quality of life at a population level. Screening programs can help detect depressive symptoms in a timely manner and reduce the no-treatment interval; however, to use available resources effectively, screening strategies should be specific and targeted. According to our previous research, and results of the epidemiological studies within the general population in other countries, the prevalence of depressive syndromes is approximately two times higher in females than in males (7, 8, 16), which leads us to consider that the risk factors of depression may differ for both sexes. For the development of effective and targeted screening strategies, we should analyze the associated factors of depression separately for males and females. After searching the available literature, there are few studies that provide information about sex-specific factors. Therefore, the aim of our study was to determine the current point prevalence of clinically relevant depressive symptoms and analyze sex-specific associated factors in a representative sample of the Latvian general adult population.

## 2. Materials and methods

We conducted a cross-sectional quantitative study based on a nationally representative, multistage stratified probability sample of 2,687 adult persons (18 years old or older) in Latvia, a country in the Baltic region of Northern Europe. At the beginning of 2019, the population of Latvia was 1.92 million people: 1.56 million of which were 18 years or older and thus formed the target population of our survey. The estimated minimal sample size required for a cross-sectional study with a randomized stratified sampling method was a total of 2,303 persons and an optimal sample size was a total of 3,595 persons. The minimal and optimal necessary number of respondents in each sex and age strata was also determined.

The starting addresses in each geographical territory were chosen from the register of State Land Service (SLS). The households to be interviewed were selected using the algorithm of the so-called random route method: starting with the initial (starting) address every second household in urban and semi-urban areas and every household in rural areas were approached. In every household only one person was interviewed according to the principle of “*Younger Man*.” If the initial contact was not made (the residents of the household were not available), the household was visited up to 3 times before being marked as not reachable. A similar random-route method has been used more than 12 times in large population-based studies in Latvia before (for example, since 1998 within the *Health Behavior of Adults in Latvia* study- a collaborative project of Estonia, Finland, Latvia and Lithuania) (17).

Data were collected through computer-assisted face-to-face interviews in Latvian or Russian, according to the respondents’ preferences. Fieldwork was conducted from November 2019 to March 2020 by 56 professional interviewers, all of whom received training sessions where the methodology, scales, and theoretical background of the study were discussed and comprehensive practical training on used instruments was provided. Up to 10 interviewers participated in each training session. The fieldwork agency followed the ESOMAR International Code on Market and Social Research, and the best professional practices of the Latvian Association of Sociologists. Electronic and audially recorded consent was obtained from participants. Since the Covid-19 pandemic started and the first Emergency state was announced in Latvia in March 2020, the field work was stopped prematurely, but we have managed to reach the minimal necessary number of valid interviews. Interviewers visited 14,506 addresses to reach the mentioned sample size. The response rate was 44.9% of selected individuals. There were no statistically significant differences in the basic sociodemographic characteristics between survey respondents and non-respondents. There were no statistically significant differences in the basic sociodemographic characteristics between survey respondents and non-respondents.

### 2.1. Measures

The questionnaire consisted of a face-to-face interview (questions about sociodemographic characteristics, alcohol use, self-rated health status, assessment of alcohol use disorder) as well as self-completion part. In order to assess depression, participants were asked to fill in the self-evaluation 9-item Patient Health Questionnaire (PHQ). The PHQ questionnaire includes the 9 symptom-based Diagnostic and Statistical Manual of Mental Disorders, Fourth Edition (DSM-IV) criteria for major depressive episode and can be used to assess the severity of depression. The PHQ-9 assesses the last 2 weeks before the interview, thus assessing the current episode or point prevalence of depression (18). This instrument has shown good sensitivity and specificity in previous research with better characteristics than other diagnostic scales (e.g., GDS-15) (19). According to the results of a recent meta-analysis, the PHQ-9 demonstrates greater sensitivity than semi-structured interviews (Structured Clinical Interview for DSM-III-R) (20). In this meta-analysis, only studies that excluded patients from psychiatric settings and those already identified as having symptoms of depression were analyzed. The PHQ-9 shows good psychometric performance both in general (21) and in specific populations, such as elderly primary care patients (22). The PHQ-9 has been used for screening purposes in specific populations, such as patients in primary care settings (23), patients with cancer (24), post-stroke depression (25), and across the general population, with proven specificity and sensitivity (7, 26). The PHQ-9 has also been used in previous studies and has been conducted in the general and primary care populations in Latvia (7, 27). Considering all of the above, the PHQ-9 has proven to be a reliable and valid instrument with the ability of categorical and dimensional analysis (28). Questions of PHQ-9 scores each of nine DSM-IV criteria as “0” (not at all) to “3” (nearly every day) and the total score ranges from 0 to 27. In our study, the presence of clinically relevant depressive symptoms was defined using a cut-off score of 10 and above. It was proven that this standard cut-off score maximizes combined sensitivity (0.85) and specificity (0.85) (29), and in our previous research on the general population, the cut-off score used was 10 points as well (7). Since, PHQ-9 do not exclude depression due to organic causes or substance misuse, in our study we use term “depression” to refer to a *clinically relevant depressive symptoms* but not as a synonym of Recurrent depressive disorder or Depressive episode, according to the International Classification of Diseases, 10^th^ edition (ICD-10) or Major depressive disorder, according to the DSM-IV. A similar terminological approach was also used in other recent international studies (23, 29, 30).

The PHQ-9 translations for both Latvians and Russians have been previously validated in primary care settings in Latvia. To assess comorbid alcohol use disorders, the corresponding module of the MINI International Neuropsychiatric Interview (M.I.N.I.), Version 7.0.2, was used. The M.I.N.I. is a brief structured screening tool that was validated by convergence with fully structured diagnostic interviews, such as the CIDI and semi-structured SCID-P (31). The good psychometric characteristics of the M.I.N.I., evidence supporting convergent and discriminant validity, brevity, and low administration cost make this interview a good instrument for epidemiological studies (32). The M.I.N.I. version used in the current study has been translated into Latvian and Russian languages by authorship holders.

### 2.2. Statistical analysis

Statistical data analyses were performed using IBM SPSS Statistics version 23 (International Business Machines Corporation’s Statistical Package for the Social Sciences). Descriptive statistics, uncorrected Pearson’s chi-square, and binary logistic regression models were used. The total and stratified point prevalence rates of depression were calculated. Due to the statistically significant difference in depression prevalence between sexes, regression analysis was performed to examine the associations between depression and each independent variable for men and women separately. Binary logistic regression was used to calculate the odds ratios (OR) for both the univariate and multivariate logistic analyses. All results are reported as OR with 95% confidence intervals (CI). Statistical significance was set at a level of 0.05.

## 3. Results

The final weighted sample included 2,687 persons (1,238 males and 1,449 females). Data were weighted by sex, age group, urbanization, region, and nationality. Table 1 summarizes the general sociodemographic characteristics of the sample. The median age of the respondents was 49.0 years (SD 18.2). The point prevalence of depression according to the PHQ-9 in the general population was 6.4% (95% CI 5.8–7.6). Prevalence of depression increased with age: 5.3% (95% CI 4.2–6.9) in the group of 18–44 years old respondents, 6.7% (95% CI 5.2–8.5) in the group of respondents aged 45–64, and 7.9% (95% CI 6.3–10.0) in the age group of 65 years and older, but the difference was not statistically significant (*p* = 0.09).

**Table 1:** Sociodemographic characteristics of the total sample (*n* = 2,687), weighted data.

Variables		*n* (%)
Sex	Male	1,238 (46.1)
	Female	1,449 (53.9)
Age (years)	18–44	1,135 (42.2)
	45–64	916 (34.1)
	>65	636 (23.7)
Ethnicity	Latvian	1,579 (58.8)
	Non-latvian	1,108 (41.3)
Marital status	Married/cohabiting	1,369 (51.0)
	Divorced/separated/widowed	784 (29.2)
	Single	534 (19.9)
Employment status	Employed	1,425 (53.1)
	Unemployed	210 (7.8)
	Economically inactive	183 (6.8)
Education	Lower than secondary	361 (13.4)
	Secondary	1,547 (57.6)
	Higher than secondary	779 (29.0)

We found that the point prevalence of depression was significantly higher among females [7.7% (95% CI 6.4–9.0)], than among males [4.8% (95% CI 4.2–6.7), *p* = 0.02]. Moreover, when adjusted for main analyzed socio-demographic characteristics (sex, age, education level, ethnicity, employment, marital status, and place of residence) simultaneously, female sex still appeared to be significantly associated with higher odds of having depression [*vs* males aOR 1.45 (95% CI 1.03–2.05), *p* = 0.03] (separate table for the mentioned regression model not shown). Taking this into account, we decided to analyze the associated factors for both sexes separately and focus on sex-specific associated factors. The point prevalence of depression among sociodemographic groups are represented for males and females in (Tables 2, 3), respectively. According to the univariate analysis for males (Table 2), the odds of having depression were significantly higher in males with only primary education [vs. higher education OR 2.3 (95% CI 1.1–4.9), *p* = 0.03], and in unemployed males [vs. employed males OR 2.3 (95% CI 1.1–4.9), *p* = 0.03]. Higher odds of depression were detected among unmarried men (vs. married or cohabiting men OR 2.1 [95% CI 1.1–3.7), *p* = 0.02] and men who were living separately, divorced, or widowed [in comparison with married males OR 2.7 (95% CI 1.4–5.3), *p* = 0.002]. Living in Latvian towns (except the capital city) appeared to be a protective factor [vs. living in rural areas OR 0.5 (95% CI 0.2–1.0), *p* = 0.04]. Similarly, protective roles had a total income of 501–900 EUR/month [vs. no income OR 0.4 (95% CI 0.1–0.9), *p* = 0.03] or income of 901 EUR/month or greater [vs. no income OR 0.3 (95% CI 0.1–0.8), *p* = 0.02]. Especially high odds of depression were detected in males who evaluated their health as “average” [vs. “good or rather good” OR 6.0 (95% CI 2.7–13.3), *p* < 0.001] and “bad or rather bad” [vs. “good” OR 25.2 (95% CI 10.9–58.4), *p* < 0.001]. According to crude analysis, alcohol use disorder (according to the M.I.N.I.) was also associated with higher odds of clinically relevant depressive symptoms [vs. no detected diagnosis OR 2.4 (95% CI 1.4–4.1), *p* = 0.001]. According to the univariate analysis for females (Table 3), higher odds of depression were found in females who completed secondary education [vs. higher education OR 1.81 (95% CI 1.05–3.14), *p* = 0.03], and especially high odds were associated with unfinished primary education [vs. higher education OR 6.2 (95% CI 2.7–13.9), *p* < 0.001]. Belonging to ethnic minorities (ethnicity other than Latvian or Russian) was also associated with a higher risk of depression [vs. Latvian OR 2.2 (95% CI 1.3–3.9), *p* = 0.006]. Economic inactivity appeared to be a predictor of depression [vs. employed status OR 1.8 (95% CI 1.2–2.8), *p* = 0.005]. As for health self-evaluation, similar tendencies, detected among males, were observed among females: women who evaluated their health as “average” [vs. good OR 3.1 (95% CI 1.8–5.2), *p* < 0.001] or as “bad/rather bad” [vs. good OR 12.2 (95% CI 7.0–21.4), *p* < 0.001] had higher odds of depression.

**Table 2:** Prevalence of depression in males, results of univariate and multivariate logistic regression analysis

			Univariate analysis			Multivariate analysis		
**Factor**	**Depression**		**OR**	**95% CI**	***p***	aOR*	**95% CI**	***p***
	***n***	**%**						
***Age***
18-44	28	4.7	1			1		
45-64	19	4.4	0.9	0.5-1.7	0.82	**0.2**	**0.1-0.6**	**0.001**
65+	13	6.1	1.2	0.6-2.5	0.52	0.4	0.1-1.3	0.11
***Education***
Higher	13	4.3	1			1		
Professional secondary	17	3.7	0.8	0.4-1.8	0.66	0.5	0.2-1.3	0.19
Secondary	13	4.6	1.1	0.5-2.3	0.86	0.7	0.3-1.7	0.40
Primary	16	9.4	**2.3**	**1.1-4.9**	**0.03**	1.6	0.6-4.1	0.32
Unfinished primary	1	5.9	0.8	0.05-11.8	0.86	0.7	0.04-12.7	0.81
***Ethnicity***
Latvian	38	5.2	1			1		
Russian	19	4.7	0.9	0.5-1.6	0.80	0.7	0.4-1.5	0.39
Other	3	3.1	0.6	0.2-1.9	0.36	0.3	0.1-1.3	0.10
***Employment***
Employed	29	3.8	1			1		
Unemployed	9	8.0	**2.3**	**1.1-5.0**	**0.03**	1.3	0.4-3.7	0.68
Economically inactive	22	6.0	1.6	0.9-2.8	0.11	0.6	0.2-1.8	0.42
***Marital status***
Married, cohabiting	24	3.3	1			1		
Unmarried	20	6.4	**2.0**	**1.1-3.7**	**0.02**	1.4	0.6-3.2	0.45
Live separately, divorced, widowed	16	8.2	**2.7**	**1.4-5.3**	**0.002**	**2.6**	**1.2-5.8**	**0.02**
***Place of residence***
Riga	23	5.3	0.8	0.5-1.5	0.60	1.1	0.5-2.4	0.79
Other city	13	3.1	**0.5**	**0.2-1.0**	**0.04**	**0.4**	**0.2-1.0**	**0.045**
Rural	24	6.1	1			1		
***Income***
No	8	10.1	1			1		
<=500 EUR/month	28	6.0	0.5	0.2-1.3	0.16	0.6	0.2-1.7	0.32
501-900 EUR/month	12	3.9	**0.4**	**0.1-0.9**	**0.03**	0.6	0.2-2.2	0.47
901+ EUR/month	10	3.4	**0.3**	**0.1-0.8**	**0.02**	0.5	0.1-1.9	0.29
***Self-rated health status***
Good or rather good	8	1.2	1			1		
Average	28	6.3	**6.0**	**2.7-13.3**	**<0.001**	**10.9**	**4.5-26.7**	**<0.001**
Bad or rather bad	24	22.4	**25.2**	**10.9-58.4**	**<0.001**	**81.9**	**27.7-241.8**	**<0.001**
***Alcohol regular use***
No	18	4.2	1			1		
Yes	42	5.2	1.2	0.7-2.1	0.53	1.4	0.7-2.8	0.37
***Alcohol use disorder according to M.I.N.I***
No	35	3.7	1			1		
Yes	25	8.5	**2.4**	**1.4-4.1**	**0.001**	1.8	0.9-3.7	0.11

* Adjusted by all factors simultaneously. Statistically significant associations marked in bold.

**Table 3:** Prevalence of depression in females, results of univariate and multivariate logistic regression analysis.

			Univariate analysis			Multivariate analysis		
**Factor**	**Depression**		**OR**	**95% CI**	***p***	aOR*	**95% CI**	***p***
	***n***	**%**						
***Age***
18-44	32	5.9	1			1		
45-64	42	8.7	1.5	1.0-2.6	0.07	0.9	0.5-1.7	0.86
65+	38	9.0	1.6	1.0-2.6	0.07	**0.4**	**0.2-0.9**	**0.02**
***Education***
Higher	27	5.7	1			1		
Professional secondary	37	7.2	1.3	0.8-2.1	0.34	1.1	0.6-2.0	0.77
Secondary	28	9.7	**1.8**	**1.0-3.1**	**0.03**	1.6	0.9-3.0	0.12
Primary	9	6.6	1.2	0.6-2.6	0.63	1.0	0.4-2.4	0.94
Unfinished primary	10	26.3	**6.2**	**2.7-13.9**	**<0.001**	**5.2**	**2.0-13.6**	**0.001**
***Ethnicity***
Latvian	55	6.5	1			1		
Russian	39	8.3	1.3	0.8-2.0	0.25	1.1	0.7-1.8	0.73
Other	18	13.2	**2.2**	**1.3-4.0**	**0.006**	1.5	0.8-2.8	0.21
***Employment***
Employed	36	5.4	1			1		
Unemployed	10	10.2	1.9	0.9-4.0	0.09	1.7	0.7-4.0	0.25
Economically inactive	66	9.6	**1.8**	**1.2-2.8**	**0.005**	**2.0**	**1.1-3.6**	**0.03**
***Marital status***
Married, cohabiting	41	6.4	1			1		
Unmarried	15	6.8	1.1	0.6-2.0	0.81	1.1	0.6-2.3	0.68
Live separately, divorced, widowed	55	9.3	1.5	1.0-2.3	0.06	1.1	0.7-2.0	0.62
***Place of residence***
Riga	45	9.5	1.3	0.8-2.1	0.29	1.5	0.8-2.6	0.17
Other city	34	6.3	0.8	0.5-1.4	0.46	0.9	0.5-1.6	0.72
Rural	33	7.5	1			1		
***Income***
No	6	8.6	1			1		
<=500 EUR/month	75	8.8	1.0	0.4-2.2	0.92	1.5	0.5-4.0	0.45
501-900 EUR/month	23	7.2	0.8	0.3-1.9	0.56	2.2	0.7-6.6	0.17
901+ EUR/month	5	4.0	0.4	0.1-1.4	0.17	2.0	0.5-8.2	0.33
**Self-rated health status**
Good or rather good	20	2.8	1			1		
Average	48	8.3	**3.1**	**1.8-5.2**	**<0.001**	**3.5**	**1.9-6.2**	**<0.001**
Bad or rather bad	44	26.7	**12.2**	**7.0-21.4**	**<0.001**	**15.6**	**7.9-30.7**	**<0.001**
**Alcohol regular use**
No	69	7.8	1			1		
Yes	43	7.7	1.0	0.7-1.5	0.93	1.3	0.8-2.1	0.27
***Alcohol use disorder according to M.I.N.I***
No	106	7.6	1			1		
Yes	6	10.0	1.3	0.6-3.2	0.52	0.9	0.3-2.5	0.89

* Adjusted by all factors simultaneously. Statistically significant associations marked in bold.

After adjustment for all independent variables for males, middle age (45–64 years) was associated with lower odds of a current depressive episode (vs. 18–44 years aOR 0.2, 95% CI 0.1–0.6), but being divorced, widowed, or living separately, in contrast, increased odds of depression [vs. being married aOR 2.6 (95% CI 1.2–5.8), *p* = 0.02]. Similarly, as in the univariate analysis, living in non-rural Latvian towns was associated with a lower risk of depression among men [vs. living in rural regions aOR 0.4 (95% CI 0.2–2.0), *p* = 0.045]. After adjustment, self-rated health status maintained its significance as a strongly associated factor of depression: odds were significantly higher in males who evaluated their health as “moderate” [vs. good aOR 10.9 (95% CI 4.5–26.7), *p* < 0.001] or “bad” [vs. good aOR 81.9 (95% CI 27.7–241.8), *p* < 0.001].

After adjusting for all independent variables for females, unfinished primary education [vs. higher education aOR 5.2 (95% CI 2.0–13.6), *p* = 0.001], as well as an economically inactive status [vs. being employed OR 2.0 (95% CI 1.1–3.6), *p* = 0.03] remained strongly associated with depression. Among females, aOR was higher in those, who considered their health to be “average” [vs. good aOR 3.5 (95% CI 1.9–6.2), *p* < 0.001] or “bad” [vs. good aOR 15.6 (95% CI 7.9–30.7), *p* < 0.001]. As mentioned above, the most significant sex-specific factors associated with depression were being divorced, widowed, or living separately for males, and poor education and economic inactivity for females.

## 4. Discussion

Our current research provides more updated information about depression in Latvia and adds a more comprehensive analysis of sociodemographic factors associated with the condition. Moreover, one of the main principles of good research is *replicability*, which implies that research outcomes can be validated by replicating research using the same or similar methodology (33). Thus, conducted in a large, representative population-based sample, our current study has some important findings that can be compared with previous epidemiological research realized with a similar methodology.

The detected point prevalence of depression (6.4%) is in line with the overall prevalence of current depressive disorder reported by other European countries (6.38%) (34) and the results of a previous Latvian epidemiological study, where the detected point prevalence of depression was 6.7% (7). The recently reported aggregate point prevalence of depression in 68 studies (19 of which have been conducted in Europe) was 12.9% (16), and, in comparison with our estimates, is higher; however, as was concluded in the above-mentioned meta-analysis, there was a high level of heterogeneity between the reported results: with statistically significant difference in the aggregate prevalence depending of used instruments, human development index of the study country and year of publication.

According to our findings, depression was significantly more prevalent among women than men. Moreover, when adjusted for all the main analyzed socio-demographic characteristics simultaneously, female sex still appeared to be significantly associated with higher odds of having depression. Similar conclusions were drawn in a recently published population-based study that analyzed data from respondents from 27 European countries (34). They found a similar tendency: the prevalence of depression was higher in females (7.74%) than in males (4.89%), with similar sex differences for all included countries, except Finland and Croatia. In a previous study conducted in Latvia, the odds of having depression were twice as high in females than males (7). There is no single simple explanation for such a pattern. The most commonly mentioned explanation is related to the different socially defined roles and challenges of women and men: women may suffer from more restricted gender roles (35), domestic violence, and pressure from expectations in school (36). Another possible explanation is that males are prone to more externalizing behaviors, such as substance abuse, while for women, more internalizing reactions, such as depression and anxiety, are more common (37, 38). Biochemical explanations can be drawn from the fact that depressive symptoms correlate with periods of hormonal changes in women (puberty, menstruation, pregnancy, and perimenopause). It is found, that moderating the cycle of estrogen may be protective for depression (39).

Our results are consistent with findings showing an increasing prevalence of depression with age; for example, in the population-based survey from our Baltic neighbor country Estonia, authors found that the odds of having depression were higher in those who were more than 45 years old (40). In an international European survey, a similar tendency was found: the highest prevalence of current depressive disorder was among those aged 75 years or older (34). On the other hand, these findings are contrary to the observation of decreased prevalence in the oldest age group from developed countries, such as the United States, where it has been found that major depression is more common in younger adults (41). A possible explanation for these opposite tendencies in Europe and the USA may include the fact that in Europe, elderly people are less economically stable than in the USA; in 2018, the annual median equivalized net income across the EU-27 was EUR 16,144 euro ($17,738) per year (42), but the median income of older persons in the USA in 2018 was $25,601 per year (43). Although the prevalence of depression varied with age in both men and women, the results of multinomial regression did not indicate age as a clear associated factor.

After adjusting for all factors simultaneously, living separately, being divorced or widowed, and poor self-rated health appeared to be the most significant factors associated with depression in men. Regarding marital status, the odds of being depressed were approximately three times higher in men who had lost marital ties (i.e., widowed, divorced, or separated). Similar results were found in a previous prevalence study in Latvia in 2011 (7)) and, for example, in a nationally representative survey in the United States (41). The more significant impact of living alone, especially on males, was detected in other studies, where authors also found that living alone had a significantly greater impact on men than on women in first onset of major depressive disorder and recurrent depression (44). One possible explanation for this finding is the assumption that women may have stronger social support from their friends, relatives, and colleagues than men, whereas men usually report their wives to be their main source of social support (45). This may explain why single, widowed, or divorced women are less vulnerable to the development of depression than men.

Poor self-rated health status was also found to be a predictive factor for depression in two previous Latvian epidemiological surveys (7, 8). In our study, poor subjective health status was associated with current clinically relevant depressive symptoms in both sexes, but in males, it was associated with 81 times higher odds of depression in comparison with subjectively healthy men. Interpreting these findings, we should take into account, that the confidence interval of estimated odds was quite wide. Since there is a small number of male respondents in the group with good self-rated health, several alternative calculations of logistic regressions were made, merging groups of good/moderate versus bad and moderate/bad versus good self-rated health, but in all of the alternative calculations the confidence levels of estimated odds remained wide, which indicates we have cannot draw a categorical conclusion about the effect of self-rated health in males, and that further information is needed.

At the same time, in females, this association was less pronounced, and poor subjective health status increased the odds of depression only 15 times, but with ordinary confidence interval. Due to the cross-sectional design of our study, we cannot draw conclusions about causality or clearly conclude which pattern of reciprocal associations between depression and poor health evaluation is dominant. According to the available research, those with existing physical diseases and poor self-rated health are at a higher risk of depression (46), but depression can also impair the subjective evaluation of health due to low self-esteem and depressed mood, the main symptoms of major depression. Moreover, objectively existing somatic disorders increase the risk of depression, which increases the risk of chronic diseases and worsens their course (47).

Interesting findings appeared after taking into consideration all the analyzed characteristics, and, as we see in Model 2, there were increased odds of depression in economically inactive women as well as women with unfinished primary education. In worldwide data, studies suggest that low levels of education may be associated with an increased risk of depression (48). Moreover, studies have also confirmed that an appropriate educational level may protect against depression, and this protective effect seems to accumulate throughout life (49). Traditional explanations of the relationship between educational level and mental health focus on the economic advantages of those with higher education (50). However, social factors may also play an important role: appropriate education provides inclusion in prosperous and cohesive social networks and structures, and, as evidence shows, social support plays a particularly important protective role for mental distress, especially among females (51). In our study, we have pointed out the importance of education for women, and, according to the available research, depression decreases more steeply for women than for men as the level of education increases (52). This allows us to consider that providing opportunities for education may lead to more prominent benefits for the mental health of women.

As for economic inactivity, available research has proven that the risk of depression is doubled in economically inactive young adults (53), but our study has specifically pointed out that women are more affected by economic inactivity (maternity leave, etc.). It is possible that economically inactive women are especially vulnerable to chronic distress and negative life events because of financial dependence, limited decision-making authority, insecure housing tenure, and reduced social support, which, as mentioned above, is especially important. In our study we have found that depression in women is not associated with economic problems in general, but specifically with economical inactivity (inability to actively earn money). Previously, there were no studies on the level of financial abuse or elements of the patriarchal form of relationships in families in Latvia, but our theoretical assumption is that it is likely that financial dependence during periods of economic inactivity is an aggravating factor for the development of mental health problems. For example, one recent study during the COVID-19 pandemic in the United States found that the percentage of women who experienced at least one form of economic violence while on maternity leave was quite high (approximately 65%) (54).

The main strength of our study is its large, nationally representative sample, which allows us to extrapolate the obtained data to the general population of Latvia. Another strength is the use of validated and internationally recognized measures for detecting depression and alcohol use disorders. Additionally, specially trained interviewers conducted the interviews and were available face-to-face to clarify questions from the self-reported part of the questionnaire. The fieldwork for the study was conducted immediately before the COVID-19 pandemic, which means that our collected data can serve as an excellent “point of reference” for subsequent studies comparing the prevalence of depression before and after the pandemic.

While interpreting and assessing the findings of this study, several methodological limitations should be considered. Because of the cross-sectional design of this study, only associations and not causation could be deduced. The use of the PHQ-9 to assess current depressive symptoms does not allow for distinguishing between major and bipolar depression, or exclusion of depression due to substance misuse and other medical conditions. Since the PHQ-9 is a self-evaluation instrument, the results can be influenced by the current situational emotional state of the respondent and subjective interpretation of symptoms. In addition, voluntary recruitment may lead to a so-called *non-response bias,* where non-responders might have different characteristics than survey respondents, which is also a considerable limitation of our study (55).

## 5. Conclusion

The point prevalence of depression in the general adult population of Latvia is 6.4% in line with other European countries and our previous research. Loss of marital ties was a significant factor associated with depression in men, and economic inactivity and unfinished primary education were more specifically associated with depression in women. Poor self-rated health status was associated with clinically relevant depressive symptoms for both sexes. These findings should be considered when developing effective and targeted screening strategies for depression. This can also be used in the planning of targeted public health interventions, such as psychological support after the loss of a marriage, especially for men, and overall welfare-promoting interventions, such as providing access to education and employment for women.

## Data availability statement

The raw data supporting the conclusions of this article will be made available by the authors, without undue reservation.

## Ethics statement

The studies involving human participants were reviewed and approved by the Ethics Committee of Riga Stradins University, Riga, Latvia (No. 6-2/8/11 from 26.09.2019). The patients/participants provided their written informed consent to participate in this study.

## Author contributions

VV, AK-U, JV, and ER conception and design of study, literature searches, analyses, and approval of the version of the manuscript to be published. JV performing training sessions for interviewers involved in field-works. VV and AK-U acquisition of data and team of 56 professional interviewers involved in field-works and analysis and interpretation of data. VV drafting the manuscript. AK-U, JV, and ER revising the manuscript critically for important intellectual content. All authors contributed to and have approved the final manuscript.

## Funding

The data collection was financed by the European Social Fund and Ministry of Health of the Republic of Latvia in the frame of the “Study on the prevalence of mental disorders and suicidal behavior in the adult population of Latvia” within the project “*Integrated measures for health promotion and disease prevention*” (Nr.9.2.4.1/16/I/001). The analysis, interpretation of data, language editing procedures, writing of the manuscript, and publication were financed by the European Social Fund and Latvian state budget within the project no. 8.2.2.0/20/I/004 “*Support for involving doctoral students in scientific research and studies*” at Rīga Stradiņš University.

## Conflict of interest

The authors declare that the research was conducted in the absence of any commercial or financial relationships that could be construed as a potential conflict of interest.

## Publisher’s note

All claims expressed in this article are solely those of the authors and do not necessarily represent those of their affiliated organizations, or those of the publisher, the editors and the reviewers. Any product that may be evaluated in this article, or claim that may be made by its manufacturer, is not guaranteed or endorsed by the publisher.
